# Rare presentation in a rare case of pancreatic extraosseous Ewing’s sarcoma: A case report

**DOI:** 10.1097/MD.0000000000031752

**Published:** 2022-11-25

**Authors:** Ya-Chih Liu, Ting-Chi Yeh, Pao-Su Wu, Jin-Cherng Sheu, Hung-Chang Lee, Chun-Yan Yeung, Chuen-Bin Jiang, Hsi-Che Liu, Jen-Yin Hou, Wai-Tao Chan

**Affiliations:** a Department of Pediatrics, Lienchiang County Hospital, Lienchiang, Taiwan; b Department of Hematology-Oncology, MacKay Children’s Hospital, Taipei, Taiwan; c Department of Medicine, MacKay Medical College, New Taipei City, Taiwan; d Department of Pathology, MacKay Memorial Hospital, Taipei, Taiwan; e MacKay Junior College of Medicine, Nursing and Management, New Taipei City, Taiwan; f Division of Pediatric Surgery, Department of Surgery, MacKay Memorial Hospital, Taipei, Taiwan; g Department of Pediatric Gastroenterology, Hepatology and Nutrition, MacKay Children’s Hospital, Taipei, Taiwan.

**Keywords:** Ewing’s sarcoma, Ewing’s sarcoma family of tumors, extraosseous Ewing’s sarcoma, pancreas

## Abstract

Extraosseous Ewing’s sarcoma is a rare tumor which is aggressive with poor prognosis; it can occur anywhere in the body, but scantily in the pancreas. Pancreatic Ewing’s sarcoma is not reported commonly, with inconsistent clinical manifestations. In this regard, early recognition of this disease is very important for the patient’s sake.

**Patient concerns:**

A 16-year-old boy presented with left lower quadrant abdominal pain for 2 months, and left flank pain with dysuria for 1 month.

**Diagnosis:**

Abdominal and renal ultrasonography found a mass between the spleen and left kidney as well as left renal pelvic dilatation. Abdominal computed tomography found a heterogenous mass derived from the tail of the pancreas. Serial examinations revealed that the mass was a pancreatic Ewing’s sarcoma. Furthermore, no metastasis was documented.

**Interventions:**

The tumor was totally excised after 6 months of chemotherapy, which included 10 courses of neoadjuvant chemotherapy with vincristine, epirubicin, and cyclophosphamide, alternating with ifosfamide and etoposide. The patient completed consolidation chemotherapy with vincristine, epirubicin, and cyclophosphamide, alternating with ifosfamide and etoposide for 5 courses. Radiotherapy was applied to the tumor-involved region and tumor bed.

**Outcomes:**

To date, the malignancy has not recurred since the treatment was completed 4 years ago. There are no complications from the treatment for the patient.

**Lessons:**

The pancreas is a very rare extraosseous location for Ewing’s sarcoma. Pancreatic extraosseous Ewing’s sarcoma should be regarded as a differential diagnosis of non-urinary originated left flank pain with dysuria in adolescents.

## 1. Introduction

Extraosseous Ewing’s sarcoma (EES) is a member of the Ewing’s sarcoma (ES) family of tumors (ESFT). It is a rare tumor of neuroectodermal origin with poor prognosis. ESFT has common morphological, histological, and genetic characteristics.^[1,2]^ EES usually occurs in children or young adults and can be found anywhere in the body, but is most commonly seen in the extremities and trunk.^[3]^ The incidence of pediatric EES is found to be 0.4 per million, while that of skeletal ES is about 10 times as that of EES.^[4]^ The pancreas is a rare location in EES. Tumors of the pancreas in young adults vary widely and include pancreatoblastoma, ductal adenocarcinoma, metastatic lesions, lymphoma, or a myriad of benign tumors.^[5]^ The treatment of these tumors is markedly different.

The patient described here is a teenager who presented with left lower quadrant abdominal pain, left flank pain, and dysuria caused by pancreatic EES.

## 2. Ethnics

Written informed consent was obtained from the patient and his mother, approved by the Institutional Review Board of MacKay Memorial Hospital (IRB number: 21MMHIS319e).

## 3. Case presentation

**Figure 1.**
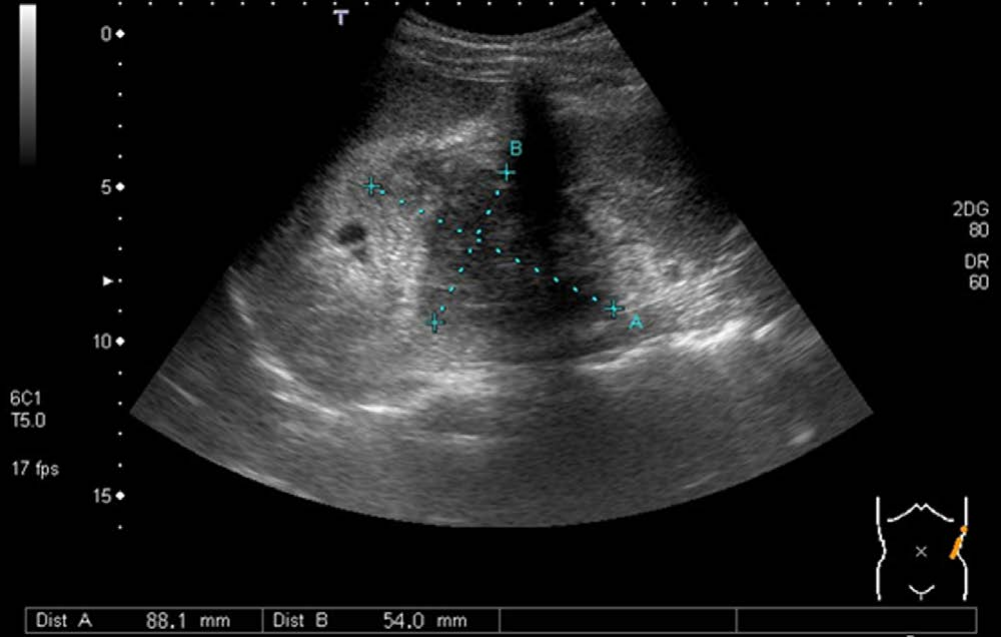
Abdominal ultrasonography. A heterogeneous mass (8.8 × 5.4 cm) was located between the spleen and left kidney with compression to the adjacent organs.

**Figure 2.**
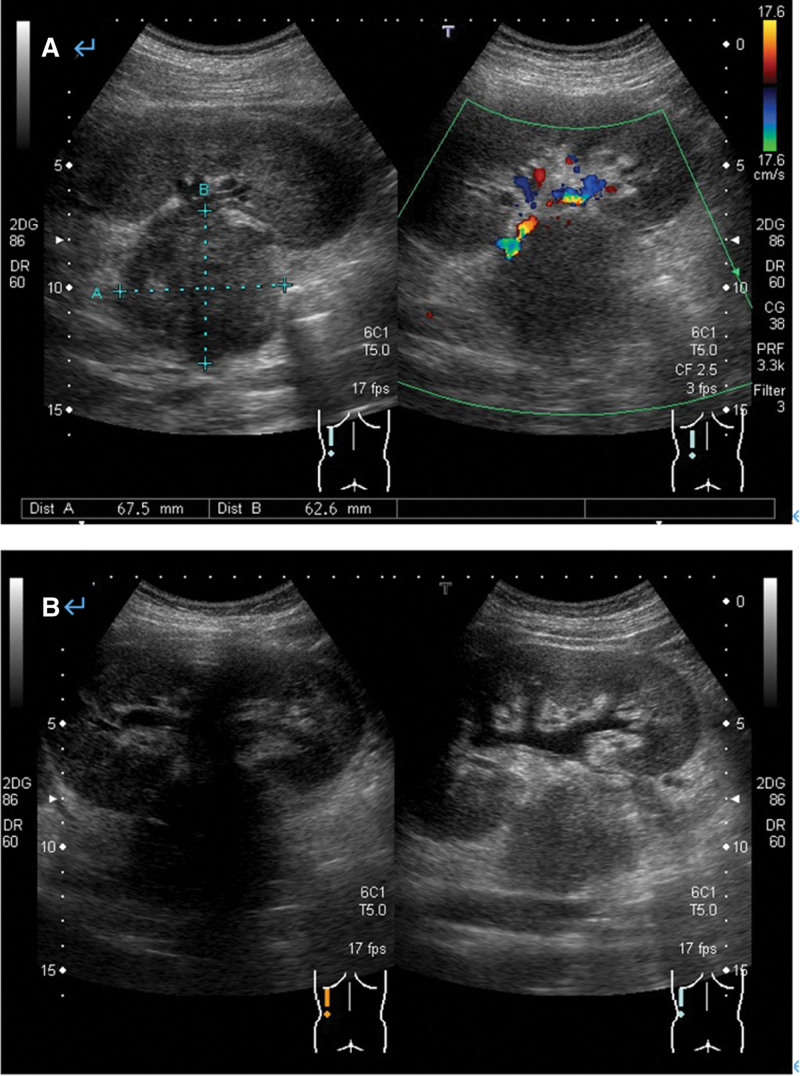
(A, B) Renal ultrasonography. A heterogeneous mass (6.75 × 6.26 cm) compressed the left renal hilum, causing left renal swelling and pelvic dilatation.

**Figure 3.**
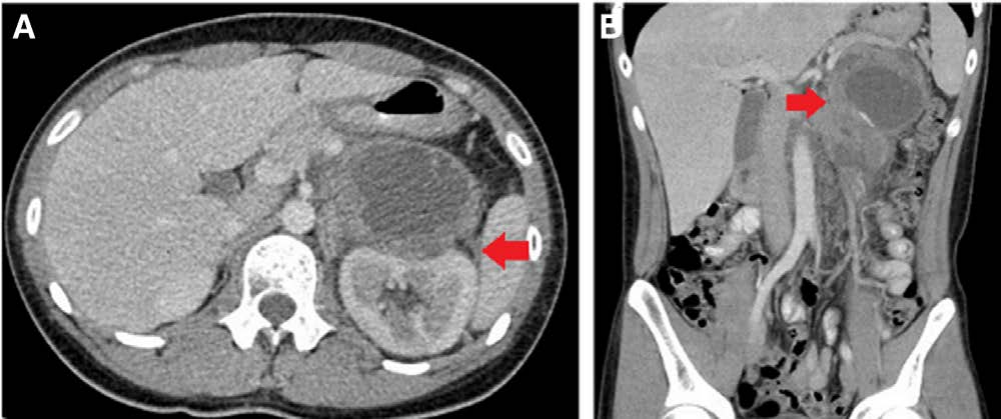
(A, B) Abdominal computed tomography. A heterogeneous mass with calcification and cystic and solid components was found in the pancreatic tail compressing the left kidney (arrow).

**Figure 4.**
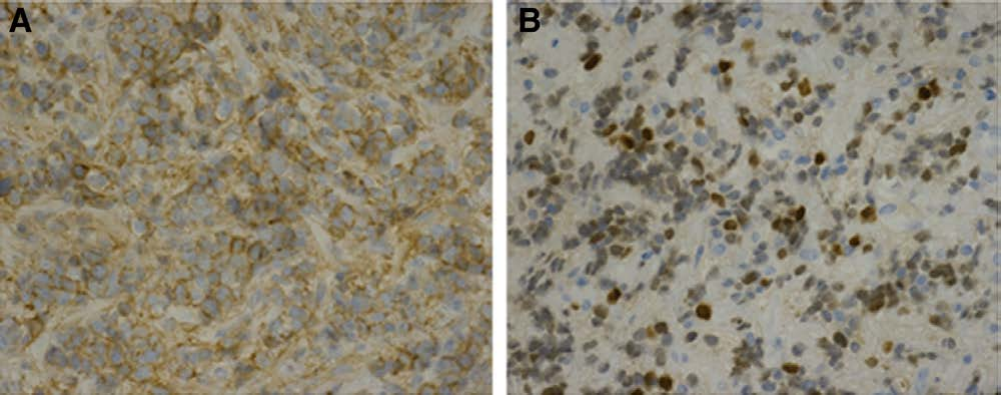
Immunohistochemical stains. Tumor cells showed complete membranous staining of CD99 (A) and nuclear staining of NKX2.2 (B).

A 16-year-old boy presented with intermittent left lower abdominal pain lasting 2 months before being presented to hospital, without diarrhea or vomiting. A low-grade fever was noted once, but subsided spontaneously. Physically, the abdomen was soft and flat, and no masses were palpated. A hemogram revealed no anemia or leukocytosis. Urine analysis did not reveal any abnormalities. However, left flank pain and dysuria developed one month later. Abdominal and renal ultrasonography (Figs. 1, 2A and 2B) showed a heterogeneous mass located between the spleen and left kidney. The mass compressed the left kidney and caused swelling and pelvic dilatation. Computed tomography (CT) of the abdomen (Figs. 3A and 3B) revealed a well-defined mass (8.0 × 5.3 × 9.3 cm) with calcified, cystic, and enhanced solid components, derived from the tail of the pancreas. Biopsy was performed. Microscopically, the sections showed tissue infiltrated with nests and sheets of small blue round cells with a high N/C ratio and inconspicuous nucleoli. Immunohistochemically (Figs. 4A and 4B), the tumor cells were positive for CD99 and NKX2.2, and negative for CD45, CK (AE1/AE3), S100 protein, myogenin, MyoD1, and WT-1. These cells retained the expression of INI-1 and the repressive histone methylation marker H3K27me3. The tumor cells were also immunopositive for FLI-1. The patient was diagnosed with Ewing sarcoma. A metastatic workup, including CT scans of the patient’s chest and brain, showed no metastasis.

**Figure 5.**
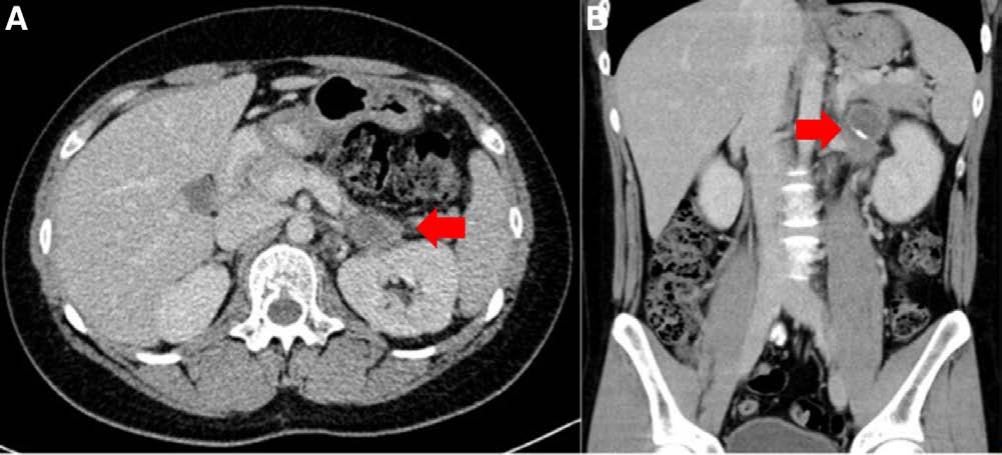
(A, B) Abdominal computed tomography. After 6 months of chemotherapy, the tumor size decreased while encasing the left renal vessels (arrow).

**Figure 6.**
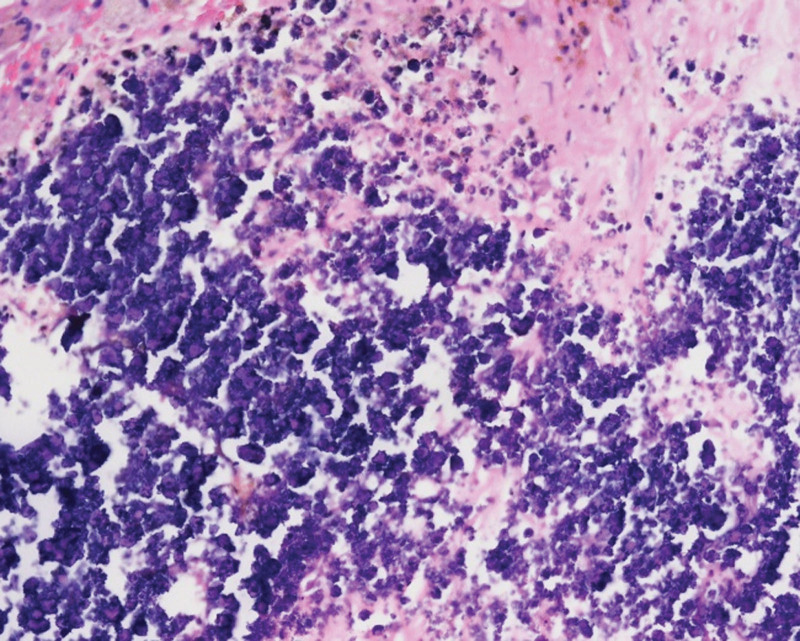
HE stains. The tumor shows complete necrosis without viable cells after chemotherapy and dystrophic calcifications are also seen.

Total excision of the tumor was performed after 6 months of chemotherapy (Figs. 5A and 5B), which included 10 courses of neoadjuvant chemotherapy with vincristine, epirubicin, and cyclophosphamide, alternating with ifosfamide and etoposide. Because the mass adhered firmly to the left renal vessels, left nephrectomy and left partial adrenalectomy were performed simultaneously. Pathological reports showed that the tumor was totally necrotic with dystrophic calcification, consistent with post-chemotherapy changes in Ewing sarcoma (Fig. 6). Immunostaining with CD99 and NKX2.2 could not identify viable tumor cells. The margins of all specimens were tumor-free. The patient has recovered well. He completed consolidation chemotherapy with vincristine, epirubicin, and cyclophosphamide, alternating with ifosfamide and etoposide for 5 more courses.

Radiotherapy was applied to the tumor-involved region and tumor bed. During the past 4 years, the patient has been under regular follow-up, and no recurrence of the tumor has been found to date.

## 4. Discussion

ES, an undifferentiated small round cell tumor first reported in 1921 by Ewing, is the second most common bone and soft tissue tumor in children.^[6–8]^ ESFT represents a family of tumors morphologically similar to small round cell neoplasms, including classic ES of the bone, EES or extraskeletal ES, soft-tissue-based primitive neuroectodermal tumors (PNETs), and small-cell tumors of the thoracic pulmonary region (Askin tumor).^[1]^

The diagnosis of ESFT includes a combination of clinical symptoms, histopathology, immunohistochemical features, and cytogenetic analysis.^[9]^ Most of the time, it is enough to diagnose ES with a core biopsy of the tumor; nevertheless, immunohistochemistry and cytogenetic analysis are essential when differentiating other small round cell tumors such as Wilms’ tumor, neuroblastoma, hepatoblastoma, rhabdomyosarcoma, small-cell lymphoma, visceral small-cell neuroendocrine carcinoma, desmoplastic small round cell tumor, pancreatic neuroendocrine tumor, and pancreatoblastoma.^[9]^

Tumors of the ES family show substantially high expression of the cell surface glycoprotein, so immunohistochemistry is helpful in diagnosing this neoplasm.^[3,10]^ This glycoprotein, a product of a pseudo autosomal gene, namely the MIC2 gene (also called CD99 or p30/32 MIC2), is found on the short arm of the X and Y chromosomes of PNETs and ESs cells. This gene is regarded as important for cell adhesion and is a marker of CD99, which is trustworthy in differentiating ESFTs from other small round cell tumors.^[3,11]^

Cytogenetic and molecular analyses are powerful tools for the classification of sarcomas. In 85-95% of cases of ES/PNETs, they show chromosome translocation at t[11;22] [q24;q12]. The second most common translocation is found at t[21;22] [q22;q12] occurs at 5% to 10%.^[8,12,13]^

EES usually originates in soft tissues and bones. In some published reports, EES may also be found in the abdominal cavity and arise from solid organs such as the kidneys, urinary bladder, ovaries, uterus, parotid glands, heart, and lungs.^[3,9]^ In addition to these organs, this type of tumor can also develop in the pancreas, accounting for 0.3% of primary pancreatic neoplasms.^[3,12]^ Aside from the case discussed here, only 26 other reports on pancreatic EES have been published in the literature.^[3,14]^

According to the literatures, the most common symptoms of pancreatic EES were as follows: abdominal pain (68%), jaundice (20%), nausea (16%), and anemia (16%).^[14]^ Endocrine disorders such as hyperglycemia and precocious puberty have also been reported in some cases.^[8]^ Pancreatic EES occurs mostly in the head of the pancreas (68%), ranging from 3.5 cm to 11 cm.^[11,12,14]^ There are few previous reports of extraosseous ES/PNET originating from the pancreatic tail.^[2]^ To our knowledge, the symptoms of flank pain and dysuria in the case reported here are first described for pancreatic EES, which may be resulted from swelling of the kidney and pelvic dilatation caused by compression of the tumor to the urinary organ.

EES are highly aggressive malignant tumors that almost inevitably recur and metastasize in the late stages. According to previous study, when diagnosed with EES, 25 to 30% of patients had already developed metastases, and the number of patients with micrometastasis may be larger.^[3]^ The standard treatment for EES is a combination of systemic multi-agent chemotherapy, surgery, and/or radiotherapy.^[8]^ Neoadjuvant chemotherapy with multi-agent chemotherapy includes vincristine, epirubicin, and cyclophosphamide alternating with ifosfamide and etoposide, followed by radical surgery, and adjuvant chemotherapy is the main treatment strategy for malignancy. This protocol improves overall survival rates and decreases the likelihood of recurrence of localized EES.^[15,16]^ The initial primary tumor size, especially a volume larger than 200cc,^[17]^ is a strong poor predictive factor of prognosis with EES. Both lung and bone metastases have poor prognosis.^[17]^ Other risk factors include older age, pelvic involvement, high white blood cell count, elevated lactate dehydrogenase level, and low hemoglobin level at the time of diagnosis.^[16]^

The overall 5-year survival rate varies depending on the condition, with or without metastatic disease, at the time of diagnosis. Patients with localized disease had 60 to 80% overall 5-year survival rates, but below 30% for patients with metastatic disease.^[3,8,9]^

Our patient was diagnosed with localized pancreatic EES. He was treated with surgical resection combined with chemotherapy and radiation therapy. Thus far, the patient has remained disease-free after treatment. Further examination and observation should be continued to detect any recurrent disease. Owing to the poor prognosis of EES, early detection of this disease is very important.

## 5. Conclusions

Pancreatic EES is a very rare tumor with a poor prognosis. Rare as it may be, pancreatic EES should be considered in the differential diagnosis of non-urinary originated flank pain and dysuria.

## Author contributions

**Conceptualization:** Ya-Chih Liu, Hung-Chang Lee, Chuen-Bin Jiang, Wai-Tao Chan.

**Data curation:** Ya-Chih Liu, Pao-Su Wu, Jin-Cherng Sheu.

**Investigation:** Ya-Chih Liu, Ting-Chi Yeh, Wai-Tao Chan.

**Methodology:** Hung-Chang Lee.

**Project administration:** Chuen-Bin Jiang.

**Supervision:** Wai-Tao Chan.

**Validation:** Hung-Chang Lee, Chun-Yan Yeung, Chuen-Bin Jiang, Hsi-Che Liu, Jen-Yin Hou.

**Visualization:** Chun-Yan Yeung, Chuen-Bin Jiang.

**Writing – original draft:** Ya-Chih Liu, Wai-Tao Chan.

**Writing – review & editing:** Ya-Chih Liu, Ting-Chi Yeh, Pao-Su Wu, Jin-Cherng Sheu, Hung-Chang Lee, Chun-Yan Yeung, Chuen-Bin Jiang, Hsi-Che Liu, Jen-Yin Hou, Wai-Tao Chan.
